# Pasos Adelante: The Effectiveness of a Community-based Chronic Disease Prevention Program^1^

**Published:** 2004-12-15

**Authors:** Lisa K Staten, Linda L Scheu, Dan Bronson, Veronica Peña, Jo Jean Elenes

**Affiliations:** Southwest Center for Community Health Promotion, Mel and Enid Zuckerman Arizona College of Public Health, University of Arizona, Division of Health Promotion Sciences; Mel and Enid Zuckerman Arizona College of Public Health, University of Arizona, Tucson, Ariz; Division of Epidemiology and Biostatistics, Mel and Enid Zuckerman Arizona College of Public Health, University of Arizona, Tucson, Ariz; Regional Center for Border Health/WAHEC, Somerton, Ariz; Platicamos Salud, Mariposa Community Health Center, Nogales, Ariz

## Abstract

**Background:**

Implementing programs that target primary prevention of chronic diseases is critical for at-risk populations. *Pasos Adelante,* or "Steps Forward," is a curriculum aimed at preventing diabetes, cardiovascular disease, and other chronic diseases in Hispanic populations. *Pasos Adelante* is adapted from the National Heart, Lung, and Blood Institute's cardiovascular disease prevention curriculum, *Su Corazón, Su Vida*, and includes sessions on diabetes and community advocacy and incorporates walking clubs.

**Context:**

The *Pasos Adelante* curriculum was implemented in two Arizona, United States-Sonora, Mexico border counties. Key issues in these communities are safety, access to recreational facilities, climate, and cultural beliefs.

**Methods:**

*Pasos Adelante* is a 12-week program facilitated by community health workers. The program includes interactive sessions on chronic disease prevention, nutrition, and physical activity. Evaluation of the program included precurriculum and postcurriculum questionnaires with self-reported measures of physical activity and dietary patterns. Approximately 250 people participated in the program in Yuma and Santa Cruz counties.

**Consequences:**

Postprogram evaluation results demonstrate a significant increase in moderate to vigorous walking among participants and shifts in nutritional patterns.

**Interpretation:**

The *Pasos Adelante* program demonstrates that an educational curriculum in conjunction with the support of community health workers can motivate people in Arizona/Sonora border communities to adopt healthy lifestyle behaviors.

## Background

The leading causes of death in U.S. Hispanic populations are heart disease/stroke, cancer, accidents, and diabetes mellitus (1). Health-compromising behaviors such as physical inactivity and poor nutrition are clearly linked to increased risk for many of these chronic diseases. Studies have demonstrated that increases in physical activity and a healthy diet can prevent or delay the onset of diabetes (1,2). This is of particular importance to individuals living along the U.S.-Mexico border, where diabetes prevalence is twice that of the rest of the nation (3). In general, U.S. Hispanics are less physically active than non-Hispanic whites (4). Although the typical eating habits of U.S. Hispanics are comparable to those of the general U.S. population, the U.S. Hispanic population consumes significantly fewer than the recommended five to nine daily servings of fruits and vegetables (5).

Effective primary prevention programs are crucial to reversing the high diabetes rates along the U.S.-Mexico border. Use of community health workers, or *promotores de salud*, is ideal for primary diabetes prevention programs that focus on lifestyle changes. *Promotores* provide enhanced community knowledge, dedication to health promotion, assistance with culturally appropriate program adaptation and supplementation, and personal knowledge of the disease experiences of their communities. Furthermore, *promotores* are shown to be effective in increasing access to care and preventive screening (6) and are frequently used in Hispanic community programs.

In 2000, the Mel and Enid Zuckerman Arizona College of Public Health received a legislative appropriation to fund a comprehensive diabetes prevention and control program, the Border Health Strategic Initiative (*Border Health ¡SI!*), in Cochise, Santa Cruz, and Yuma, Arizona counties. The *Border Health ¡SI!* program included behavioral intervention components targeting providers (7), people with diabetes (8), their families (9), the general community, and school environments (10). The program also included a policy component (11). For a detailed discussion of *Border Health ¡SI!*, read the overview by Cohen et al (3).

This paper describes the community component, *Pasos Adelante*, or "Steps Forward." The purpose of the project was to collaboratively develop and implement a community-based chronic disease prevention program and to demonstrate the effectiveness of the program in reducing risk factors for cardiovascular disease, diabetes, and other chronic diseases related to diet and physical activity. This article provides details about the unique communities involved, the participatory development and evaluation of the program between community organizations and university personnel, and the community and university perspectives about the value and impact of the program.

## Context

The *Pasos Adelante* program was implemented in southwestern Yuma County and southern Santa Cruz County, Arizona, from 2000 to 2003. The communities share a border with Mexico. While each is unique in its economy, employment rates, demographic structure, and ethnic composition, as described by Cohen et al (3), the population of both communities is predominantly Hispanic, with high rates of poverty and unemployment. Various factors within these communities may contribute to health-compromising behaviors, including the availability of recreational facilities, climate, nutrition, and cultural beliefs.

There are virtually no recreational facilities, walking paths, or safe areas in which to exercise in these communities. Many neighborhoods do not have sidewalks or crosswalks. Additional concerns identified over the project period included lack of lighting and roaming dogs. The extreme heat acts as another barrier to physical activity. Temperatures range from an average low of 29°–41° F in December to 96°–106° F in July.

A survey conducted in 1998 in a neighboring Arizona border community reported a diabetes prevalence of 18%, obesity or overweight in 74%, and no regular physical activity among 67.4% of respondents (12). Many additional factors contribute to this high rate of overweight. Healthy food choices such as low-fat milk are not often readily available in rural communities (13). Because 63% to 78% of the population reports incomes less than 200% of the federal poverty level (14), many people do not have the resources to purchase the frequently more expensive healthier items.

An additional barrier to promoting healthy lifestyle choices may be cultural beliefs. Some participants expressed beliefs that people can develop diabetes by having it wished upon them or by putting the body through hot and cold extremes. These participants may not be receptive to the idea that physical activity and a healthy diet will in any way affect their chances of developing diabetes. Other cultural factors must be addressed also, such as the belief that walking is indicative of low socioeconomic status and therefore not desirable.

## Methods

### Formative phase

Two key concepts of the *Border Health ¡SI!* were partnership and collaboration between community partners and university personnel, as well as use of the *promotor(a)* model whenever appropriate. At the onset of *Border Health ¡SI!*, a need was identified to develop and implement a program facilitated by *promotores* to educate community members about diabetes and what can be done to prevent or delay its onset. The key partners in the development and implementation of the community component were Mariposa Community Health Center (MCHC) in Nogales, Ariz, and Regional Center for Border Health/Western Arizona Health Education Center (WAHEC) in Somerton, Ariz, with technical assistance from the University of Arizona (UA). Both community agencies have had award-winning *promotor(a)* programs for more than a decade.

During the formative phase of *Border Health ¡SI!*, diabetes prevention programs implemented by the two community agencies were documented and reviewed. Simultaneously, a comprehensive search for culturally appropriate prevention programs was performed to take advantage of existing curricula. *Promotores* and staff members from the two agencies suggested using *Su Corazón, Su Vida*, a cardiovascular disease prevention manual developed for Latinos by the National Heart, Lung, and Blood Institute (NHLBI) (15). After reviewing other curricula, the partners agreed to adapt the NHLBI curriculum to include diabetes. They chose the NHLBI curriculum for several reasons. First, the curriculum was developed by a trusted source. Second, cardiovascular disease is a major complication of diabetes and is therefore an appropriate part of a diabetes prevention program. Third, the partners supported the idea of expanding the target audience beyond persons at risk for diabetes. Fourth, most other curricula identified focused on patients with diabetes and not on disease prevention. Fifth, the curriculum was viewed as culturally competent; it targets the appropriate level of literacy and is available in Spanish. Finally, the curriculum was designed to be facilitated by *promotores*, and a number of them had previous training in the curriculum and liked the implementation style.

The main drawback to *Su Corazón, Su Vida* was that it did not focus on diabetes. A second drawback was the lack of a published program evaluation. Because *Border Health ¡SI!* would be adapting and evaluating the program, however, these concerns were not viewed as strong reasons not to use it.

### Curriculum design

To take advantage of the *Su Corazón, Su Vida* curriculum, the manual was modified to broaden the focus to include an emphasis on diabetes prevention. Two sessions addressing diabetes and a single session on community health advocacy were created and inserted. Diabetes prevention materials used by the two community agencies were also incorporated. The revised curriculum is called *Pasos Adelante.*

The *Pasos Adelante* curriculum (available from: URL: http://www.borderhealthsi.org/steps_pasos.htm*) consists of a manual and free-standing flip charts created for *Pasos Adelante* in addition to a flip chart, telenovela magazine, and video available from *Su Corazón, Su Vida*. The *Pasos Adelante* curriculum was written in both English and Spanish, although it was only implemented in Spanish in our two communities. It follows the *Su Corazón, Su Vida* scripted teaching style; the *promotores* were given a script to follow if they desired, thereby enhancing the consistency of each session among *promotores* and from group to group. Additional background information was included in each session so that *promotores* were prepared for more in-depth questions about each topic. The manual consists of 12 two-hour sessions (Figure). The sessions  "Are you at risk for diabetes?," "Glucose and sugar," and "Is your community healthy?" were designed for the *Pasos Adelante* curriculum. Each session consists of five main components: an introduction, the session in action, a weekly promise, a review of the day's most important points, and the close of the session. Furthermore, as part of the session in action, participants engage in a physical activity, such as dancing or aerobics, to reinforce the importance of physical activity.

**Figure:** *Pasos Adelante* program session topics in Spanish and English (from introductory handout).

**Table:** 

**Sesiones de Pasos Adelante**	

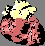	**1 **	**¿Está usted en riesgo de desarrollar enfermedades del corazón? **
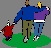	**2**	**Manténgase físicamente más activo.**
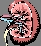	**3 **	**¿Está usted en riesgo de desarrollar la diabetes?**
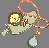	**4 **	**Todo lo que necesita saber acerca de la presión arterial alta, la sal y el sodio.**
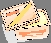	**5 **	**Coma menos grasa, grasa saturada y colesterol.**
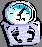	**6 **	**Mantenga un peso saludable.**
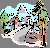	**7 **	**Nuestra comunidad, ¿es saludable? **
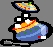	**8 **	**La glucosa y el azúcar.**
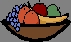	**9 **	**Goce con su familia de comidas saludables para el corazón. **
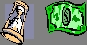	**10 **	**Coma más saludable por su corazón — aun cuando tenga poco tiempo o dinero.**
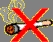	**11 **	**Goce de la vida sin el cigarrillo.**
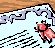	**12 **	**Repaso y graduación.**

**Steps Forward Sessions**	

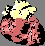	**1 **	**Are you at risk for heart disease?**
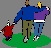	**2**	**Be more physically active.**
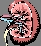	**3 **	**Are you at risk for diabetes?**
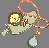	**4 **	**What you need to know about high blood pressure, salt, and sodium.**
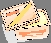	**5 **	**Eat less fat, saturated fat, and cholesterol.**
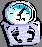	**6 **	**Maintain a healthy weight.**
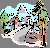	**7 **	**Is our community healthy?**
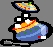	**8 **	**Glucose and sugar.**
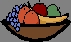	**9 **	**Make healthy eating a family affair.**
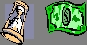	**10 **	**Eat healthier — even when time or money is tight.**
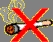	**11 **	**Enjoy living smoke-free.**
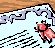	**12 **	**Review and graduation.**

In addition to weekly classroom sessions, walking clubs were incorporated into the *Pasos Adelante* program. The walking clubs were designed to engage participants in recreational walking in a coordinated, socially supportive effort to increase physical activity. The walking club was designed so that participants would initially walk together outside of class for at least 20 minutes once a week with the *promotor(a)* at a mutually agreed-upon location, such as a park or local school track. Gradually, the group would build up to walking at least 20 minutes three times a week. At week seven of the program, the *promotores* start to withdraw from the groups but continue to encourage them during class sessions so that the groups can be self-sustained after the program ends. Walking clubs were incorporated into all *Pasos Adelante* sessions to move from the didactic focus of physical activity into actual behavior change. Staff and *promotores* from MCHC and WAHEC and UA personnel met monthly to discuss curriculum development.

### Facilitator training

MCHC and WAHEC were contracted to implement the *Pasos Adelante* program. Each agency hired or reorganized existing *promotores* to participate in the *Border Health ¡SI!* program. The selection of *promotores* was left entirely to the agencies. Nine *promotores* employed by the two centers participated in curriculum design and received approximately six hours of manual training. During this training, evaluation instruments and protocols were also discussed and reviewed. All discussions and trainings were conducted in Spanish. Two additional *promotores* were hired during the project period and were trained individually by other *promotores* with technical assistance from UA personnel.

During the training, *promotores* were encouraged, but not required, to use the script. Emphasis was placed on the content and flow of each session. If *promotores* were unsure of themselves, they tended to rely on the script. Those who were comfortable making public presentations preferred a less formal style. Additional training was conducted when necessary throughout the program.

In addition to attending the *Pasos Adelante* training, many *promotores* had attended week-long trainings for *Su Corazón, Su Vida* at an annual community health worker conference cosponsored by WAHEC and therefore understood the fundamental design of the *Pasos Adelante* program. *Promotores *who had not attended *Su Corazón, Su Vida *training prior to starting the *Pasos Adelante *program did so during *Pasos Adelante *implementation. All *promotores* worked in pairs, with senior *promotores* paired with junior *promotores*. In addition, the *promotores* attended a variety of trainings on diabetes, including *Diabetes: La Comunidad en Accion,* sponsored by the Diabetes Today National Training Center and Diabetes Training for Lay Health Workers, sponsored by MCHC.

### Program implementation

Eleven *promotores* (10 women, one man) led the sessions working in pairs. (The one male *promotor* facilitated one 12-week session of the program.) Sessions were scheduled for two-hour periods but ranged from 90 minutes to 150 minutes. At times the physical activity portion at the end of the class was eliminated to complete the educational portion.

To address some of the previously identified barriers, classes were conducted at centrally accessible public locations, such as schools, churches, the MCHC, and other public multipurpose rooms. One agency provided onsite childcare services. If necessary, participants were encouraged to bring their children or grandchildren. Class members decided the times of the class and walking groups. In the Yuma area, where the weather is the most extreme, the walking groups frequently met around 5:00 AM or in the late evening to avoid the heat. The *promotores* indicated that long-term residents did not have problems with walking so early or late, but newer residents did. According to the *promotores*, long-term residents regularly used those hours to avoid the heat.

### Process evaluation

The community partners were essential to adapting and developing the *Pasos Adelante* manual. The *promotores* provided feedback on sessions and walking clubs using program-specific feedback forms. The feedback forms asked if information was missing and whether the information made sense, was adequate, and was presented in a style and manner easily understood by the group. After both agencies had completed one 12-week session, a meeting was held with all *promotores* to discuss what worked and what did not work. The *promotores* praised the curriculum and offered some minor grammatical corrections but little constructive criticism. Although the UA personnel were gratified to hear that the *promotores *liked the program, they were skeptical of the response and afraid that the *promotores* might have a cultural bias against expressing anything that sounded like criticism. So a second strategy for feedback was developed. All the manuals of the *promotores* were collected and examined. We found extensive notes in the margins of the manuals and additional handouts indicating where more information or clarification was needed. These notes allowed us to initiate a more direct conversation on the *promotores'* interpretation of the materials: "You wrote in the margin that. . . . Would you like to share with us what you mean?" This enabled us to avoid putting responsibility on the *promotores* for pointing out problems.

UA personnel were occasionally able to observe the sessions and meet with *promotores* afterwards. UA personnel contributed feedback on the actual presentation of the material and on effective communication styles. They also offered additional information, if needed.

### Recruitment

Program participants represented a convenience sample recruited by *promotores* through presentations at schools, church groups, internal agency programs, and health fairs and by going door-to-door. Classes were offered year round from 2001–2003, except during holidays. Group size averaged 10 to 15 participants.

After a consent form was signed, each participant was asked to answer a standardized physical activity risk-assessment questionnaire to ensure that the individual was physically able to participate without any serious physical or medical risk. If an individual indicated any risk, he or she was then required to obtain a provider's permission to participate. One site offered screenings for all participants.

Participants then completed a questionnaire consisting of nutrition questions based on the Centers for Disease Control and Prevention's Behavioral Risk Factor Surveillance Survey (BRFSS) (16) and physical activity questions targeting moderate to vigorous activities using a one-month format adapted from the Minnesota Leisure Time Physical Activity Questionnaire (17). The BRFSS and adapted Minnesota questionnaire have been used among low-income Hispanic populations and have been shown to be reliable indicators of behavior. The intake form was designed to identify changes in the frequency and exertion levels of moderate to vigorous physical activity and in dietary consumption patterns that would reflect curriculum content. The questionnaire focused on changes in frequency in weekly consumption of fruit, vegetables, and sweet beverages. It also asked about the changes in the type of milk consumed and the type of fat used for cooking. Questionnaires were repeated at 12 weeks (the end of class).

### Statistical methods

Intercooled STATA 7.0 (StataCorp LP, College Station, Tex) was used to analyze the quantitative data. McNemar chi-square tests were used for categorical data, Wilcoxon rank sum tests were used for independent continuous data, and Wilcoxon sign ranked tests were used for paired continuous data. Nonparametric tests were used because variables were not normally distributed. Matched pairs *t*-tests were calculated to compare age and body mass index (BMI). Significance was assigned at *P* ≤ .05. Because of small sample size, trends are identified if *P* ≤ .10.

## Consequences

A total of 248 participants began the program and 216 completed it, for a completion rate of 87%. The participants who completed the program were mainly Hispanic women born in Mexico who had not graduated from high school (Table 1). The average participant age was 49.5 years. Compared to those who completed the program, the 32 individuals who did not complete the program were significantly more likely to be employed full- or part-time and to have asthma; more smokers were among those who did not complete the program.

As Table 2 shows, self-reported changes were found in levels of physical activity and in nutrition from preclass to postclass. The number of participants walking and the number of minutes per week of moderate to vigorous walking significantly increased. There were significant reductions in the weekly consumption of sweetened soda and sweetened hot drinks and an increase in the consumption of fruit juice. The number of servings of salads, vegetables, and fruits eaten per week also increased significantly.

Results differ between the two sites. Fewer significant changes were seen in Santa Cruz participants than in Yuma area participants. For example, in Santa Cruz, only the average number of minutes walking at a moderate pace and the average number of salads eaten per week increased significantly. These differences may be due to slight demographic differences, as shown in Table 1. Santa Cruz participants were significantly younger (45.1 ± 15.5 years vs 52.7 ± 13.4 years) and more educated; fewer were born in Mexico, and more had health insurance.

In addition to statistical evidence of positive changes, *promotores* frequently commented on seeing people walking and observing that some were losing weight. Anecdotal comments were overheard or recorded in the end-of-session evaluation sheets. For example:

"A person commented in my class that for the first time in her life she is walking for 15 minutes.""One woman said that she felt really embarrassed to go out and walk, so she didn't. Now she's happy because she walks and feels comfortable doing it.""One woman explained that she would eat a whole can of corn not imagining the number of portions and amount of sodium in it. She's going to pay more attention.""One woman said she has lost six pounds since the beginning of the class. She is very happy. Also, her mother's blood sugar levels have dropped."

## Interpretation

One of the key findings from this project is that while it is difficult to get people walking when the temperature is extremely high or when no sidewalks exist, it is not impossible. Residents in the Yuma area were not reluctant to walk during the summer months. Many residents have been farm workers and are used to an early-morning lifestyle. Participants were also able to make changes in their diet. Many of the participants initially indicated that they had no idea how to eat healthier.

The formal evaluation of community-based programs is difficult. Without funds to support more systematic evaluation, organizations frequently rely on self-reported data. Our evaluation instrument was feasible and effective for our agency. It provided statistical evidence for positive changes that at the very least indicate an increased awareness of healthy lifestyle behaviors among participants. The self-reported increases in walking matched the observations of the *promotores*. They report seeing a number of their participants continuing to walk without them. The *promotores* also report individuals losing weight and participants telling them that their providers are happy with their health improvements.

Another important outcome was the integration of the *Pasos Adelante* program and the Special Action Groups, community-based coalitions formed as part of the *Border Health ¡SI!* model (11). *Promotores* reported to the coalitions monthly to quarterly about issues raised during their class sessions. Based on reports from the *promotores*, the coalitions worked to have parks, playgrounds, and walking paths incorporated into city development plans. The creation of a new park in the Yuma area resulted directly from the coalition network and a motivated *promotora*. The Yuma coalition also targeted grocery stores as arenas for promoting and increasing the availability of healthier food options.

One of the drawbacks of this program was its lack of male participants. The *Pasos Adelante* program is based on the theoretical foundation of social support, including organized group activities to promote physical activity. Men may not be as responsive as women to programs emphasizing social support. In the future, it is important to determine how the *Pasos Adelante* program could be tailored to appeal to a male audience. Additionally, the program reached a generally older population. In communities where more than half the population is under the age of 35, programs need to be developed that target primary prevention and appeal to younger people.

The *Pasos Adelante* program has demonstrated that an educational curriculum in conjunction with the support of *promotores* can motivate people to adopt healthy lifestyle behaviors. The integration of classroom sessions and walking clubs allowed for increased interactions among participants and helped create social support for nutritional and physical behavioral change. In areas with abundant educational programs and sources of information, these kinds of programs may not result in behavior change; however, in areas with relatively few resources where residents have not repeatedly been exposed to prevention messages, these programs may have much greater impact.

Other communities can use the *Pasos Adelante* curriculum. Educational sessions have occurred with *promotores* in Mexico, and work has begun on the adaptation of the curriculum for a Native American health department. Prior to implementing the program, we would suggest that an advisory committee of local community members review the curriculum and decide what changes should be made to ensure that it is culturally appropriate. It is critical that the review committee include individuals who are truly part of the target community and share its cultural beliefs.

## Figures and Tables

**Table 1:** Demographic and Health Status Comparison by Community of Pasos Adelante Participants (n=216) Who Completed Preprogram and Postprogram Assessments, Arizona, 2000–2003^a^

**Characteristic**	**Yuma County ** **Community** **N=128**	**Santa Cruz County ** **Community** **N=88**	***P***

**Demographics**			

**Age, mean (SD)**	52.7 (13.4)	45.1 (15.5)	<.001
**Hispanic**	127 (99.2)	88 (100)	.40
**Female**	113 (88.3)	84 (96.5)	.03
**Marital status**			
Married	96 (80.7)	55 (67.9)	.002
Single/divorced	10 (8.4)	22 (27.2)	
Widowed	13 (10.9)	4 (4.9)	
**Education**			
Some elementary	88 (69.8)	26 (29.9)	<.001
Elementary	27 (21.4)	25 (28.7)	
Some high school	7 (5.6)	26 (29.9)	
High school	4 (3.2)	10 (11.5)	
**Currently employed**	24 (19.0)	12 (13.8)	.23
**Health insurance**	40 (32.3)	61 (69.3)	<.001
**Preferred speaking language is Spanish**	121 (96.8)	76 (97.4)	.80
**Preferred reading language is Spanish**	122 (97.6)	76 (96.2)	.56
**Length in community**			
<5 years	27 (21.1)	21 (23.1)	.03
>10 years	84 (65.6)	54 (62.1)	
**Born in Mexico**	124 (97.6)	75 (87.2)	.003

**Health Status**			

**Diagnosed with diabetes**	21 (16.4)	24 (27.6)	.06
**How long been a diabetic**			
<1 year	4 (19.1)	8 (36.4)	.43
1-5 years	8 (38.1)	4 (18.2)	
6-10 years	3 (14.3)	4 (18.2)	
10+ years	6 (28.6)	6 (27.3)	
**Family member diagnosed with diabetes**	53 (41.4)	54 (62.1)	.002
**Participant with a heart condition**	6 (4.7)	10 (11.5)	.17
**High cholesterol**	37 (29.1)	24 (27.3)	.93
**Diagnosed with cancer**	6 (4.7)	3 (3.4)	.66
**Diagnosed with osteoporosis**	12 (9.5)	5 (5.8)	.21
**Diagnosed with asthma**	1 (0.8)	4 (4.6)	.07
**Current smoker**	4 (3.1)	7 (8.0)	.11

a Values are numbers (percentages) unless otherwise indicated. Percentages are based on the number of participants who responded to the question; not all participants (n=216) responded to every question.

**Table 2:** Preprogram and Postprogram Comparison of Self-reported Physical Activity and Dietary Intake, Pasos Adelante Participants (n=216), Arizona, 2000–2003

	**n^a^**	**Median (range)**	**Mean (SD)**	***P***

**Physical Activity**				

**Fast walking, minutes/week**	198			.002
Preprogram		0 (0-1800)	77.5 (±204.5)	
Postprogram		0 (0-780)	108.9 (±160.0)	
**Moderate walking, minutes/week**	191			<.001
Preprogram		0 (0-840)	73.7 (±117.7)	
Postprogram		120 (0-840)	138.10 (±145.4)	
**Slow walking, minutes/week**	202			.81
Preprogram		0(0-840)	45.7 (±107.1)	
Postprogram		0(0-420)	40.5 (±82.1)	

**Dietary Intake **				

**Soda, servings/week**	204			<.001
Preprogram		0.5(0-49)	2.6 (±5.7)	
Postprogram		0(0-21)	1.4 (±2.9)	
**Diet soda, servings/week**	204			.20
Preprogram		0(0-35)	1.7 (±4.7)	
Postprogram		0(0-35)	1.7 (±4.3)	
**Sweetened drink, servings/week**	208			.24
Preprogram		2(0-56)	4.7 (±7.5)	
Postprogram		2(0-28)	4.3 (±5.8)	
**Sports drink, servings/week**	203			.07
Preprogram		0(0-28)	1.6 (±3.9)	
Postprogram		0(0-21)	1.0 (±2.4)	
**Sweetened hot drink, servings/week**	205			.03
Preprogram		7(0-49)	7.5 (±7.8)	
Postprogram		7(0-28)	6.5 (±6.0)	
**Fruit juice, servings/week**	205			.01
Preprogram		3.7(0-42)	5.8 (±6.0)	
Postprogram		7(0-28)	6.6 (±5.7)	
**Salad, servings/week**	208			<.001
Preprogram		3(0-35)	4.5 (±5.2)	
Postprogram		5.5(0-28)	6.2 (±5.1)	
**Vegetables, servings/week**	205			<.001
Preprogram		3(0-21)	5.3 (±5.0)	
Postprogram		7(0.25-28)	7.8 (±5.9)	
**Fruits, servings/week**	204			<.001
Preprogram		7(0-49)	8.8 (±7.3)	
Postprogram		7(0-49)	11.7 (±7.9)	
**Fruits and vegetables, servings/week**	201			<.001
Preprogram		12(0-63)	14.2 (±9.7)	
Postprogram		16(3-63)	19.6 (±11.8)	

a n = number of participants who answered the question in both the preprogram and postprogram questionnaire. Not all participants (n=216) responded to every question. SD = standard deviation.
